# Furthering the idea of proportionate governance in British Columbia

**DOI:** 10.23889/ijpds.v1i1.391

**Published:** 2017-04-19

**Authors:** Kim Mcgrail

**Affiliations:** 1 University of British Columbia

## Objective

In British Columbia today, every request for access to data for research purposes is subject to the same time-consuming and intensive review. This presents challenges for timeliness of reviews and scalability as the current process is equally labour intensive for a simple request for limited data, and a request where there is intent to contact. This approach also undermines the public’s interest in supporting research that has potential public value.

Building on the work of SHIP, we have developed a proportionate risk review framework that is intended to make access decisions transparent, expand the potential users of data beyond university-based researchers, and enable a broader range of inquiry. Adding to this external goals, this presentation will describe the use of this framework for internal audit purposes.

## Approach

Six proposed core principles of review are science, approach, data, people, environment, and interest, with a spectrum of risk for each ranging from low to very high. The principles and spectrum of risk create a grid, which represents all the possible scenarios under which access to data may be sought and provides a visual means of rating and assessing applications. A significant challenge for the implementation of the framework is identifying consensus on what constitutes `low risk' and `high risk' and mapping risk profiles to a clear review process. This will be pursued using deliberative engagement approaches to public consultation.

## Results

In the meantime, in order to build understanding of and support for the framework with data stewards, we have employed it to track and monitor service provision to requesting researchers. We identified both "quick" and "slow" projects as examples of researcher experience with data access. Retrospectively, we mapped those projects to the framework to show their levels of risk. We identified the length of time taken at each stage of the request process, from developing an application to receipt of data. We used the proportionate risk framework and information on speed of approval to generate discussion about what factors of review are most concerning to data stewards. Through this we have the ability to institute new policies and come closer to implementing a formal proportionate review process.

## Conclusion

The proportionate risk review framework is a flexible tool that can be used to help internal processes as well as improving transparency in the data access process more generally.

